# Cigarette Smoking as a Risk Factor of Coronary Artery Disease and its Effects on Platelet Function

**DOI:** 10.1186/1617-9625-2-1-27

**Published:** 2004-03-15

**Authors:** Teruo Inoue

**Affiliations:** 1 Department of Cardiology, Koshigaya Hospital, Dokkyo University School of Medicine, Koshigaya, Saitama, Japan

## Abstract

It has been well established that cigarette smoking is a powerful risk factor for coronary artery disease. A number of epidemiologic studies have shown a strong association between cigarette smoking and atherosclerosis, myocardial infarction and death from coronary artery disease. In addition to active smoking, passive smoking can also carry a risk of coronary artery disease. Although the detailed mechanism through which cigarette smoking is associated with cardiovascular disease has not yet been clarified, it is suggested that cigarette smoking is related to thrombogenesis, as well as atherogenesis, and blood platelet behavior is thought to be prominent among the proposed mechanisms involved in atherogenesis and thrombogenesis. The following is a review of evidence that cigarette smoking affects platelet function.

## Platelet Activation by Cigarette Smoking

### Platelet aggregation studies

The most common approach to studying the effect of cigarette smoking on platelet function has been to measure the platelet aggregation response to agonists ex vivo, e.g. adenosine diphosphate (ADP), collagen, thrombin, platelet activating factor, etc. The relationship between platelet aggregation and cigarette smoking was examined by a large number of studies. These studies suggest that smoking has two effects on platelets, i.e., a significant acute potentiation of platelet activation occurring shortly after smoking a cigarette, and a chronic desensitization of the cell to activating agents occurring during the period between cigarettes. Although there are numerous longitudinal investigations of the acute effects of smoking on platelet aggregation, the results produced have been conflicting. Some have demonstrated increased platelet aggregability immediately after smoking [1-4] while others have found no such change [5-7]. Blache and colleagues [4] observed that platelet aggregation to thrombin and ADP increased significantly 10 minutes after inhalation of one cigarette smoke. However, Siess and colleagues [5] demonstrated that cigarette smoke inhalation did not enhance platelet aggregation stimulated by ADP, epinephrine, and collagen. On the other hand, regarding the effects of chronic smoking, there have also been conflicting reports of increased, decreased and unaltered platelet aggregation response to agonists ex vivo by conventional platelet aggregometric methods [8-10]. The cross sectional results from the Caerphilly Collaborative Heart Disease Study [11] show that the ADP-induced platelet aggregation response was increased among habitual smokers, but the finding was mainly seen in the subjects who had smoked cigarettes shortly before venisection.

Conversely, Foo and colleagues [9] demonstrated that in habitual smokers platelet aggregability to aggregating agents was reduced, compared with non-smokers. Lassila and colleagues [10] demonstrated that platelet aggregation responses to ADP and epinephrine at rest were similar in non-smokers and smokers but that after submaximul exercise the responses were less in the smokers than in the non-smokers. From these results of reduced platelet aggregability in habitual smokers it has been suggested that in chronic smokers, increased generation of platelet agonists in vivo might result in platelet receptor down-regulation and thus, aggregation response to these agonists is depressed ex vivo. However, conventional platelet aggregation studies are inherently limited in the information they can provide concerning the effects of smoking on platelet aggregation in vivo. Recently, however, platelet aggregability has been assessed by newly developed systems to closely evaluate platelet aggregation.

A conventional aggregometor using optical density provides only information about large aggregates of platelets, because small aggregates may be formed when platelet-rich plasma is stirred at 37 degrees C in the absence of chemical stimulants [12]. In contrast, a particle-counting technique using laser light scattering makes it possible to separately detect small, medium and large aggregates. Fusegawa and colleagues [13] compared the differences in platelet aggregability between 90 healthy male smokers and 141 age-matched nonsmoking healthy males, using this technique. In spontaneous aggregation, small aggregates were higher in smokers than in nonsmokers. Detection of medium and large aggregates formed by 1 or 5 μM of epinephrine and all aggregates by 1 μM of epinephrine were significantly higher in smokers than in nonsmokers. Aggregation induced by 5 μM of ADP showed no significant difference in small, medium, and large aggregates between the groups (Table 1). Smokers showed a positive correlation between age and 1 μM epinephrine-induced large platelet aggregates (R = 0.43, P < 0.001) and 1 μM ADP-induced medium (R = 0.30, P < 0.01) and large aggregates (R = 0.32, P < 0.01). Smokers also showed a positive correlation between fibrinogen concentration in plasma and small spontaneous aggregates (R = 0.37, P < 0.001). On the other hand, nonsmokers showed a significant positive correlation between age and small spontaneous aggregates (R = 0.32, P < 0.01), a positive correlation between fibrinogen and 1 (R = 0.23, P < 0.01) or 5 μM (R = 0.28, P < 0.01) epinephrine-induced large aggregates, and between 1 μM ADP-induced large aggregates (R = 0.24, P < 0.01). These results suggest that platelet aggregability evaluated by small aggregates is enhanced in habitual smokers, and that long-term smoking enhances the sensitivity of platelets to epinephrine or ADP.

**Table 1:** Comparison of platelet aggregation between smokers and nonsmokers

	**Smokers**	**Nonsmokers**	**P**
Spontaneous aggregation			
Small aggregates (10^5 ^counts × 10 second)	129 ± 170	62 ± 105	<0.0001
Epinephrine 1 mm			
Small aggregates (10^5 ^counts × 10 second)	523 ± 253	491 ± 218	
Medium aggregates	227 ± 146	153 ± 144	<0.0001
Large aggregates	202 ± 201	135 ± 184	0.0015
Epinephrine 5 mm			
Small aggregates (10^5 ^counts × 10 second)	346 ± 217	383 ± 227	
Medium aggregates	232 ± 122	190 ± 98	0.0318
Large aggregates	350 ± 222	278 ± 212	0.0466
ADP 1 mm			
Small aggregates (10^5 ^counts × 10 second)	592 ± 269	458 ± 243	0.0005
Medium aggregates	169 ± 180	108 ± 148	0.0025
Large aggregates	139 ± 201	91 ± 184	0.0007
ADP 5 mm			
Small aggregates (10^5 ^counts × 10 second)	236 ± 123	247 ± 140	
Medium aggregates	154 ± 58	156 ± 65	
Large aggregates	529 ± 185	486 ± 184	

**Figure 1:**
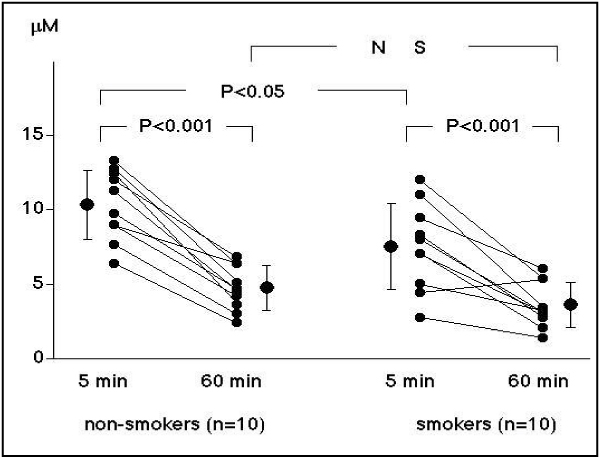
**Time-dependent changes in PATI in non-smokers (left) and smokers (right)**. In both subject groups, PATI was decreased at 60 minutes after blood sampling. The PATI values at 5 minutes after blood sampling were significantly lower in the smokers than in the non-smokers, while the values at 60 minutes were identical in both subject groups [25].

**Figure 2:**
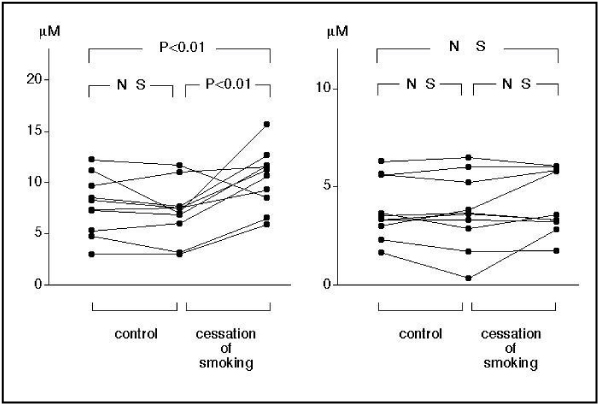
**Effect of 4 weeks' cessation of smoking on platelet aggregability**. PATI at 5 minutes after blood sampling increased after cessation of smoking (left), although there was no change in the PATI at 60 minutes after blood sampling (right).

Measurement of platelet aggregability using conventional aggregometry requires making platelelet-rich plasma, so results can be obtained about 60 minutes after blood sampling. In contrast, a novel type platelet aggregometor, the WBA analyzer (SSR Engineering, Yokohama, Japan) is a system based on a screen filtration pressure (SFP) method, which measures the resistance of the flow of whole blood samples containing platelet aggregates through a micromesh filter with 300 square openings of 20 × 20 μm. Since this system requires only 5 minutes to obtain platelet aggregation results, we can obtain the data immediately after blood sampling, and this can be considered to reflect in vivo platelet function more closely. Using this system, platelets were stimulated by various concentrations of ADP. The filtration pressure raised by clogging of the filter by aggregatory clots was measured as an index of platelet aggregation. The percent aggregation rate was calculated in each ADP concentration by the maximum filtration pressure as 100% aggregation. An ADP concentration producing 50% aggregation was calculated as a platelet aggregatory threshold index (PATI) [25]. We measured the platelet aggregatory threshold index (PATI) 5 minutes after blood sampling and compared it with that 60 minutes after blood sampling in 20 healthy male volunteers (10 smokers and 10 non-smokers) [25]. In the non-smokers, PATI was 10.3 ± 2.3 μM 5 minutes after blood sampling, decreasing to 4.7 ± 1.5 (P < 0.001) 60 minutes after blood sampling. In the smokers, the PATI was 7.7 ± 2.9 μM 5 minutes after blood sampling, decreasing to 3.8 ± 1.5 (P < 0.001) at 60 minutes after blood sampling (Figure 1). In the smokers, the PATI 5 minutes after blood sampling increased after 4 weeks' cessation of smoking (10.4 ± 2.9, P < 0.01), although the PATI 60 minutes after blood sampling did not change (4.2 ± 1.6 μM) (Figure 2). These results suggest that, in habitual smokers, platelet aggregability is enhanced immediately after blood sampling and platelets are constantly activated in-vivo.

### Other platelet function studies

An alternative approach to the study of platelet function in vivo has been that of radiolabelled platelet turnover studies. A shorter platelet lifespan has been observed in chronic smokers [15,16]. While platelet turnover studies provide definitive evidence for platelet function abnormality in chronic smokers, they do not discriminate between the direct effect of smoking on platelets and altered platelet function caused by smoking-induced vascular damage. Although studies of platelet turnover provide the opportunity to study platelet kinetics in vivo, these studies have several intrinsic limitations; they provide conclusions that are highly model-dependent and do not lead themselves to repeated application within persons.

Platelet specific proteins, platelet factor 4 (PF-4) and β-thromboglobulin (β-TG), are released from platelet α-granules into circulating blood plasma when platelets are activated. Elevated plasma levels of PF-4 [17] and β-TG [18] have been reported in the blood of chronic smokers, and the levels are also enhanced as an acute effect of smoking [4]. To investigate in-vivo platelet activation states, flow-cytometric analysis of the activation-dependent platelet membrane surface glycoproteins, including P-selectin [19-21] or activation-dependent neoepitope of glycoprotein (GP) IIb/IIIa [20,22], is available. P-selectin is a component of the α-granule membrane of resting platelets and is expressed on the platelet surface membrane after α-granule secretion when platelets are activated [19]. A soluble isoform of P-selectin shedded from the platelet surface can be also measured in the blood plasma [23].

Higher P-selectin expression on the surface of platelets and a higher plasma soluble P-selectin level in chronic smokers than non-smokers was demonstrated and thus increased in-vivo platelet activation was confirmed [24]. A chronic effect of cigarette smoking on platelet activation has also been shown by a few studies measuring elevated excretion of a thromboxane A2 metabolite (TX-M) in urine by gas chromatographymass spectrometry [25,26]. FitzGerald and coworkers [27] demonstrated the platelet origin of TX-M by a platelet-selective low dose of acetylsalicylic acid (ASA), which blocks the cyclooxygenase enzyme. The increased smoking-induced excretion of TX-M could be masked with ASA for the survival time of platelets. Platelets have an L-argininenitric oxide (NO) pathway through constitutive NO synthase (NOS) in human platelets. Platelet aggregation is inhibited by L-arginin, a precursor of NO, and potentiated by N^G^-monomethyl-L-arginine (L-NMMA), an inhibitor of NOS. Platelet aggregation is acompanied by an increase in the intracellular level of cyclic guanosine 3', 5'-monophosphate (cGMP) [28]. These findings indicate evidence of the functional L-arginine-NO pathway in platelets during aggregation, which acts as a negative feedback mechanism to inhibit not only platelet activation [29], but also recruitment after aggregation [30]. Recently, it has been shown that impaired platelet-derived NO (PDNO) production may contribute to the pathophysiology of acute coronary syndrome [31]. In chronic smokers, the bioactivity of PDNO is impaired, and it is considered that this impairment may be caused by an imbalance in the intraplatelet redox state through increased oxidative stress in smokers [32].

### Passive smoking and platelet activation

"Passive smoking" or "environmental tobacco smoke (ETS)" is the term used to characterize tobacco combustion products inhaled by non-smokers in the proximity of burning tobacco. Most ETS exposure is from sidestream smoke emitted from the burning tip of the cigarette. Non-smokers chronically exposed to ETS are believed to assume health risks similar to those of a light smoker. Several epidemiological studies have shown that the risk of ischemic heart disease is about 30% greater in non-smokers who live with smokers than in those who do not [33-35]. Although environmental exposure to cigarette smoking is only about 1% that of smoking, the risk is nearly half of the active smoker [36].

An earlier study demonstrated that the relationship between passive smoking and the higher risk of hemostatic imbalance [37]. Acute exposure to passive smoke induced in non-smokers a short-lasting activation of platelet function and the prostaglandin system, followed by a quick recovery after only 15 minutes' exposure to passive cigarette smoke. Six hours after exposure no changes were detected. Repeated exposure of non-smokers to passive smoke, however, resulted in a continuous change in the basal values for platelet aggregation, while circulating endothelial cells and microaggregates were similar but less severe. Platelet proteins were less altered. Changes were comparable to those seen in smokers, characterized by an activation of platelet function, namely, a decrease in platelet sensitivity and binding sites to the antiaggregatory prostagrandin (PG) I_2_.

In one experiment, non-smokers and smokers were asked to smoke two cigarettes [38]. The smokers' platelets, which were less sensitive to exogenous PGI_2 _than the non-smokers' platelets at the beginning of the experiment, did not significantly change their activity in response to the two cigarettes. Most likely, the smokers' platelets were already desensitized to antiaggregatory PGI_2 _because of chronic exposure to the toxins in cigarette smoke, so the addition of a relatively small (compared with what a smoker receives continuously) amount of toxins in two cigarettes had no additional effects. In contrast, smoking just two cigarettes significantly decreased non-smokers' platelet sensitivity to PGI_2_, to the point that it was not significantly different from that of habitual smokers. These data demonstrate that the responses of non-smokers and smokers to toxins in the cigarette smoke are often very different.

In another experiment that more closely parallels the experience of non-smokers [38], platelet sensitivity to PGI_2 _was measured in smokers and non-smokers before and after they sat in a room for 20 minutes where cigarettes had been smoked just before the experimental subjects entered. Again, there was no significant change in the sensitivity to PGI_2 _among the smokers, but a significant decrease in the sensitivity to PGI_2 _among the non-smokers, to the point that their platelet sensitivity to PGI_2 _was not discernibly different from that of the smokers. These data indicate that non-smokers are much more sensitive to tobacco smoke exposure than smokers, and that very low levels of ETS exposure can have a major impact on non-smokers' platelet activity. Actually, Davis and colleagues [39-43] observed a decrease in the platelet aggregation ratio (0.87 to 0.78; P = 0.002), which reflects an increased formation of platelet aggregates, and found that passive smoking increased platelet platelet aggregation with a magnitude similar to that observed in active smoking.

It also appears that the process saturates at low doses; once the non-smoker has been exposed to even a low dose of ETS, the platelets are maximally activated, similar to a habitual smoker. These data also indicate that dose-based extrapolations from smokers to non-smokers using "cigarette equivalents" will grossly underestimate the risks to non-smokers of breathing second-hand smoke.

Animal data also support this conclusion. In studies done to evaluate the effects of ETS on heart disease, it has been found that bleeding time, another measure of platelet activity, is significantly shortened in both rabbits [44,45], and rats [46] exposed to low doses of ETS, with no additional effects at higher doses. These findings indicate activated platelet function after ETS exposure. Apparently, the acute response is more pronounced in non-smokers than in smokers. On repeated exposure to cigarette smoke, platelet function in non-smokers approaches that of smokers.

## Conclusion

Cigarette smoking is a major risk factor for cardiovascular morbidity and mortality and is a leading cause of death. The effect of cigarette smoking on coronary risk factors is pervasive. Unfavorable effects include enhancement of platelet function. Platelet activation by cigarette smoking is linked to thrombosis formation, including onset of myocardial infarction. In light of the adverse effects on platelet function, cessation of smoking should be encouraged.

## Competing interests

The authors declare that they have no competing interests.

## References

[B1] Levine PH (1973). An acute effect of cigarette smoking on platelet function: – a possible link between smoking and arterial thrombosis. Circulation.

[B2] Renaud S, Blanche D, Dumont E, Thevenon C, Wissendanger T (1984). Platelet function after cigarette smoking in relation to nicotin and carbon monoxide. Clin Pharmacol Ther.

[B3] Schmidt KG, Rasmussen JW (1984). Acute platelet activation induced by smoking – in vivo and ex-vivo studies in humans. Thromb Heamost.

[B4] Blache D, Bouthillier D, Davignon J (1992). Acute influence of smoking on platelet behaviour, endothelium and plasma lipids and normalization by aspirin. Atherosclerosis.

[B5] Siess W, Lorenz R, Roth P, Weber PC (1982). Plasma catecholamines, platelet aggregation and associated thromboxane formation after physical exercise, smoking or norepinephrine infusion. Circulation.

[B6] Laszlo E, Kaldi N, Kovacs L (1983). Alteration in plasma proteins and platelet functions with aging and cigarette smoking in helthy men. Thromb Haemost.

[B7] Pittilo RM, Clarke JMF, Harris D (1984). Cigarette smoking and platelet adhesion. Br J Haematol.

[B8] Beswick A, Renaud S, Yarnell JWG, Elwood PC (1991). Platelet activity in habitual smokers. Thromb Haemost.

[B9] Foo LC, Roshidah I, Aimy MB (1991). Platelets of habitual smokers have reduced susceptibility to aggregating agents. Thromb Haemost.

[B10] Lassila R, Laustiola KE (1988). Physical exercise provokes platelet desensitization in men who smoke cigarettes: involvement of sympathoadrenergic mechanisms: a study of monozygotic twin pairs discordant for cigarette smoking. Thromb Res.

[B11] Elwood PC, REnaud S, Sharp DS, Beswick AD, O'Brien JR, Yarnell JWG (1991). Ischemic heart disease and platelet aggregation – the Caerphilly Collaborative Heart Disease Study. Circulation.

[B12] Fusegawa Y, Goto S, Handa S, Kawada T, Ando Y (1999). Platelet spontaneous aggregation in platelet-rich plasma is increased in habitual smokers. Thromb Res.

[B13] Fusegawa Y, Handa S (2000). Platelet aggregation induced by ADP or epinephrine is enhanced in habitual smokers. Thromb Res.

[B14] Inoue T, Hayashi M, Uchida T, Takayanagi K, Hayashi T, Morooka S (2001). Significance of platelet aggregability immediately after blood sampling and effect of cigarette smoking. Platelets.

[B15] Mustard JF (1963). Effect of smoking on blood coagulation and platelet survival in man. Br Med J.

[B16] Fuster V, Chesboro JH, Frye R, Elveback LR (1983). Platelet survival and the development of coronary artery disease in the young adult: effects of cigarette smoking, strong family history and medical therapy. Circulation.

[B17] FitzGerald GA, Pedersen AK, Patrono C (1983). Analysis of prostacyclin and thromboxane A2 biosynthesis in cardiovascular disease [Editorial]. Circulation.

[B18] Dotevall A, Kutti J, Teger-Nilsson AC, Wadenvik H, Wihelmsen L (1987). Platelet reactivity, fibrinogen and smoking. Eur J Haemotology.

[B19] Stenberg PE, McEver RP, Shuman MA, Jaques YV, Bainton DF (1985). A platelet alpha-granule membrane protein (GMP-140) is expressed on the plasma membrane after activation. J Cell Biol.

[B20] Inoue T, Sohma R, Morooka S (1999). Cilostazol inhibits the expression of activation-dependent membrane surface glycoprotein on the surface of platelets stimulated in vivo. Thromb Res.

[B21] Nair S, Kulkarni S, Camoens HMT, Ghosh K, Mohanty D (2001). Changes in platelet glycoprotein receptors after smoking – a flow cytometric study. Platelets.

[B22] Shattil SJ, Hoxie JA, Cunningham M, Brass LF (1985). Changes in the platelet membrane glycoprotein IIb-IIIa complex during platelet activation. J Cell Biol.

[B23] Gearing AJH, Newman W (1993). Circulating adhesion molecules in disease. Immunol Today.

[B24] Pernerstorfer T, Stohlawetz P, Stummvoll G, Kapiotis S, Szekeres T, Eichler HG, Jilma B (1998). Low-dose aspirin does not lower in vivo platelet activation in healthy smokers. Br J Haematol.

[B25] Fischer J, Bernutz C, Meier H, Weber PC (1986). Formation of prostacyclin, and thromboxane in man as measured by the main urinary metabolites. Biochim Biophys Acta.

[B26] Nowak J, Murray JJ, Oates JA, FitzGerald GA (1987). Biochemical evidence of a chronic abnormality in platelet and vascular function in healthy individuals who smoke cigarettes. Circulation.

[B27] FitzGerald GA, Oates JA, Nowak J (1988). Cigarette smoking and hemostatic function. Am Heart J.

[B28] Radomski MW, Palmer RMJ, Moncada S (1990). An L-arginine/nitric oxide pathway present in human platelets regulates aggregation. Proc Natl Acad Aci USA.

[B29] Moncada S, Palmer RMJ, Higgs EA (1991). Nitric oxide: physiology, pathphysiology, and pharmacology. Pharmacol Rev.

[B30] Freedman JE, Mickelson AM, Barnard MR (1997). Nitric oxide release from activated platelets inhibit platelet recruitment. J Clin Invest.

[B31] Freedman JE, Ting B, Hankin B (1998). Impaired platelet production of nitric oxide predicts presence of acute coronary syndrome. Circulation.

[B32] Takajo Y, Ikeda H, Haramaki N, Murohara T, Imaizumi T (2001). Augumented oxidative stress of platelets in chronic smokers. J Am Coll Cardiol.

[B33] Glanzs SA, Parmley WW (1991). Passive smoking and heart disease: epidemiology, physiology and biochemistry. Circulation.

[B34] Wells AJ (1994). Passive smoking as a cause of heart disease. J Am Coll Cardiol.

[B35] Kritz H, Schmid P, Sinzinger H (1995). Passive smoking and cardiovascular risk. Arch Intern Med.

[B36] Law MR, Morris JK, Wald NJ (1997). Environmental tobacco smoke exposure and ischaemic heart disease: an evaluation of the evidence. Br Med J.

[B37] Sinzinger H, Virgolini I (1989). Are passive smokers at greater risk of thrombosis?. Wien Wochenschr.

[B38] Burghuber OC, Punzengruber C, Sinzinger H, Harber P, Slberbauer K (1985). Platelet sensitivity to prostacyclin in smokers and non-smokers. Chest.

[B39] Davis JW, Shelton L, Watanabe TS, Arnold J (1989). Passive smoking affects endothelium and platelets. Arch Intern Med.

[B40] Davis JW, Hartmann CR, Lewis HD, Shelton L, Eigenberg D, Hassanein K, Hignite C, Ruttinger H (1985). Cigarette smoking-induced enhancement of platelet function: lack of prevention by aspirin in men with coronary artery disease. J Lab Clin Med.

[B41] Davis JW, Shelton L, Eigenberg D, Hignite C, Watanabe I (1985). Effects of tobacco and non-tobacco cigarette smoking on endothelium and platelets. Clin Pharmacol Ther.

[B42] Davis JW, Shelton L, Hartmann CR, Eigenberg D, Ruttinger H (1986). Smoking induced changes in endothelium and platelets are not affected by hydroxyethylrutosides. Br J Ekp Pathol.

[B43] Davis JW, Shelton L, Eigenberg D (1987). Lack of effect of aspirin on cigarette smoke-induced increase in circulating endothelial cells. Haemostasis.

[B44] Zhu B-Q, Sun Y-P, Sievers R, Isenberg R, Glanz SA, Parmley WW (1993). Passive smoking increases experimental atherosclerosis in cholesterol-fed rabbits. J Am Coll Cardiol.

[B45] Sun Y-P, Zhu B-Q, Sievers R, Glanz SA, Parmley WW (1994). Metoprolol does not attenuate atherosclerosis in lipid-fed rabbits exposed to environmental tobacco smoke. Circulation.

[B46] Zhu B-Q, Sun Y-P, Sievers R, Glanz SA, Parmley WW, Wolfe CL (1994). Exposure to environmental tobacco smoke increases myocardial infarct size in rats. Circulation.

